# Invasive Group A Streptococcal Infection in Older Adults in Long-term Care Facilities and the Community, United States, 1998–2003^1^

**DOI:** 10.3201/eid1312.070303

**Published:** 2007-12

**Authors:** Michael C. Thigpen, Chesley L. Richards, Ruth Lynfield, Nancy L. Barrett, Lee H. Harrison, Kathryn E. Arnold, Arthur Reingold, Nancy M. Bennett, Allen S. Craig, Ken Gershman, Paul R. Cieslak, Paige Lewis, Carolyn M. Greene, Bernard Beall, Chris A. Van Beneden, for the Active Bacterial Core surveillance/Emerging Infections Program Network

**Affiliations:** * Centers for Disease Control and Prevention, Atlanta, Georgia, USA; † Minnesota Department of Health, Minneapolis, Minnesota, USA; ‡ Connecticut Department of Public Health, Hartford, Connecticut, USA; § Johns Hopkins Bloomberg School of Public Health, Baltimore, Maryland, USA; ¶ Georgia Department of Human Resources, Atlanta, Georgia, USA; # University of California at Berkeley School of Public Health, Berkeley, California, USA; ** University of Rochester School of Medicine and Dentistry, Rochester, New York, USA; †† Tennessee Department of Health, Nashville, Tennessee, USA; ‡‡ Colorado Department of Public Health and Environment, Denver, Colorado, USA; §§ Oregon State Public Health, Portland, Oregon, USA; 1 Presented at the 42nd Annual Meeting of the Infectious Diseases Society of America, September 30–October 3, 2004, Boston, Massachusetts, USA.

**Keywords:** group A streptococcus, surveillance, elderly, long-term care facilities, mortality, research

## Abstract

Limited information exists on the incidence and characteristics of invasive group A streptococcal (GAS) infections among residents of long-term care facilities (LTCFs). We reviewed cases of invasive GAS infections occurring among persons >65 years of age identified through active, population-based surveillance from 1998 through 2003. We identified 1,762 invasive GAS cases among persons >65 years, including 1,662 with known residence type (LTCF or community). Incidence of invasive GAS infection among LTCF residents compared to community-based elderly was 41.0 versus 6.9 cases per 100,000 population. LTCF case-patients were 1.5 times as likely to die from the infection as community-based case-patients (33% vs. 21%, p<0.01) but were less often hospitalized (90% vs. 95%, p<0.01). In multivariate logistic regression modeling, LTCF residence remained an independent predictor of death. Additional prevention strategies against GAS infection in this high-risk population are urgently needed.

## Abstract 2

Invasive infection develops almost 6 times as frequently in the elderly in long-term care facilities.

Although group A *Streptococcus* (GAS) most commonly causes pharyngitis and soft tissue infections (*1*), it also produces severe invasive disease including bacteremia, pneumonia, necrotizing fasciitis (NF), and streptococcal toxic shock syndrome (STSS), especially at the extremes of age (*2*,*3*). In the United States, 9,000–11,000 cases and 1,100–1,800 deaths from invasive GAS infection occur each year (*3*). Those >65 years of age have the highest incidence and case-fatality rate: nearly a third of all cases and half of all deaths occur in this age group (*3*). In addition to advanced age, cardiac and vascular disease, diabetes, skin breakdown, corticosteroid use, and malignancy are associated with increased risk for invasive GAS infection among adults (*4*–*8*). Because underlying conditions are common among long-term care facility (LTCF) residents, this population may be especially vulnerable to invasive GAS infection. Although outbreaks of invasive GAS infections have been well described among LTCF residents (*9*–*16*), the extent and characteristics of sporadic invasive GAS infections in this population have not been well defined.

Since 1998, the Active Bacterial Core surveillance (ABCs) of the Emerging Infections Program Network (EIP)—a collaboration between the Centers for Disease Control and Prevention (CDC), state health departments, and academic centers—has collected information on residence (LTCF vs. community) of invasive GAS case-patients. We used ABCs data to compare incidence, characteristics, and factors contributing to death from invasive GAS infections of elderly LTCF residents and similar-aged persons residing in the community.

## Methods

### Surveillance

ABCs conducts active laboratory- and population-based surveillance for invasive infections due to GAS and other bacterial pathogens of public health importance. We reviewed ABCs reports of invasive GAS cases among persons >65 years of age occurring from January 1, 1998, through December 31, 2003, in the following sites: San Francisco, California (3 counties); Baltimore, Maryland (6 counties); Albany and Rochester, New York (15 counties); Portland, Oregon (3 counties); Chattanooga, Knoxville, Memphis, and Nashville, Tennessee (11 counties); and the entire states of Connecticut, Georgia, and Minnesota. Five counties in the Denver, Colorado, metropolitan area were added in 2000. The total surveillance area encompassed a 2000 Census population of 3,446,404 persons >65 years of age (10% of the total US population in this age group).

ABCs methodology has been published previously (*2*,*17*). Briefly, ABCs sites maintain active contact with clinical laboratories to identify all cases and perform audits of laboratory records at least every 6 months to ensure complete reporting. Surveillance officers review case-patient medical records to obtain information on demographic characteristics, clinical syndrome, underlying disease, and illness outcome. Case-patients with GAS-positive blood cultures but without an identifiable clinical syndrome are categorized as having bacteremia without focus. Otherwise, multiple clinical syndromes—including cases of pneumonia, cellulitis, osteomyelitis, non-skin abscesses, and other syndromes (*18*) when accompanied by a sterile site isolate—may be reported for each case. Underlying illness information (*18*) was not consistently collected in Georgia from 1998–1999, Maryland from 1998–2000, or Tennessee in 1998. Information on smoking status was collected beginning in 2000 and history of cerebrovascular accident (CVA) in 2001.

### Case Definitions

ABCs defines a case of invasive GAS infection as isolation of GAS from a normally sterile site (e.g., blood, cerebrospinal fluid) or from a wound when accompanied by STSS or NF in a resident of an ABCs surveillance area. ABCs defines an LTCF as a skilled nursing facility, nursing home, rehabilitation hospital, or other chronic-care facility in which the patient has been living for at least 30 days before GAS infection. The definition did not include facilities in which the patient receives daily outpatient therapy or prisons, group homes, and assisted living facilities.

To determine whether outbreaks contributed significantly to GAS disease among LTCF residents, we looked for clusters within LTCFs. We defined a GAS LTCF cluster as >2 invasive infections with the same *emm* type occurring within 12 months (duration of some previously reported GAS outbreaks [*10*]) among residents >65 years of age living at the same facility. Surveillance staff confirmed the residence of case-patients within each cluster.

### Descriptive Epidemiology

To describe incidence trends for persons >65 years of age (regardless of residence type) from 1998 to 2003, we analyzed GAS cases and deaths reported from 54 ABCs counties that conducted GAS surveillance during the entire 6-year period (1998 population: 1,981,662 persons >65 years of age). For annual rate calculations, we used national census and postcensus population estimates for these counties as the annual population denominators.

To calculate incidence of invasive GAS infection among persons >65 years of age stratified by residence type, we included ABCs GAS case-patients during the year 2000 and imputed cases with missing residence information on the basis of distribution of cases with known residence. For the denominator we used residence type–specific population estimates from the US Census 2000 Summary File 1 for ABCs counties (*19*); census data on residence type were only available for the year 2000. To calculate national estimates of disease, we applied age- and race-specific GAS rates from the ABCs surveillance area to the age and racial distribution of the US population in 2000; we redistributed those of unknown race on the basis of the reported distribution for known cases.

For residence-specific analyses, we excluded cases of invasive GAS infection if residence was missing or unknown. To calculate case-fatality ratios (CFRs) we included only case-patients with known outcomes.

### Microbiologic Testing

ABCs sites forwarded all available GAS isolates to CDC’s Streptococcal Genetics Laboratory. GAS isolates underwent T typing and amplicon restriction profiling of the *emm* gene as described at www.cdc.gov/ncidod/biotech/strep/protocol_emm-type.htm (*20*). Using a reference database containing ≈180 group A streptococcal *emm* sequence types, we categorized an isolate as a given *emm* type if it had >92% identity over the first 30 codons encoding the processed M protein with one of the reference *emm* types (*21*).

Antimicrobial drug susceptibility testing of available GAS isolates in 1999, 2001, and 2003 was performed at CDC by using broth microdilution. To report antimicrobial susceptibility, we used established Clinical and Laboratory Standards Institute breakpoints for MICs and defined isolates with intermediate or high-level resistance as nonsusceptible (*22*).

### Statistical Analysis

We used SAS version 9.1 (SAS Institute Inc., Cary, NC, USA) for all analyses. To analyze incidence trends, we used Cochran-Armitage calculations for linearity and trend. In univariate analysis, we used Cochran-Mantel-Haenszel statistics to compare case-patient and GAS isolate characteristics stratified by case-patient residence; we also analyzed factors associated with death among LTCF residents and community-based case-patients separately.

We used logistic regression to characterize factors associated with death, checking for 2-way interactions and collinearity. We included in our model all variables associated with death on univariate analysis (p<0.15) controlling for age group, race, and sex. We stratified *emm* type into each of the 10 most common *emm* types and an 11th category including all remaining *emm* types (“other”). We classified case-patients with multiple clinical syndromes in the category with the highest CFR. The model was restricted to cases for which information on all variables was available. We considered p values <0.05 statistically significant.

## Results

### Disease Incidence and Estimated Disease Impact in the Elderly

From 1998 to 2003, a total of 5,889 cases of invasive GAS infection of all ages were reported, including 1,762 (30%) among persons >65 years of age. Incidence of invasive GAS infection in this elderly age group increased from 10.0 cases per 100,000 population in 1998 to 10.9 cases per 100,000 population in 2003 (Table 1). Type of residence was available for 1,662 elderly case-patients (94%). Of these, 383 case-patients resided in LTCFs, accounting for 23% of cases in those >65 years of age. In 2000 (the only year with reliable US Census population estimates for residence type), the incidence of invasive GAS among LTCF residents was almost 6 times higher than among community-based residents (41.0 vs. 6.9 cases per 100,000 persons, p<0.01). Projecting to the US population, we estimate that 650 cases among LTCF-residents and 2,250 cases among community-based residents >65 years of age occurred nationwide in 2000. Among both LTCF- and community-based residents, GAS incidence was highest among black men (78.9 and 13.8 cases per 100,000 persons, respectively) and lowest among white women (35.1 and 4.9 cases per 100,000 persons, respectively).

**Table 1:** Invasive group A streptococcal infection cases and deaths among persons age >65 y, by site, ABCs areas, 1998–2003*

	1998	1999	2000	2001	2002	2003
No. cases/100,000 population						
CA	8.4	10.3	11.1	9.8	7.6	9.5
CT	8.7	9.4	11.3	9.8	10.2	11.5
GA	10.5	7.3	9.7	12.5	6.4	9.5
MD	13.7	9.0	9.3	15.4	11.4	15.3
MN	11.4	10.5	10.6	13.1	10.3	9.8
NY	7.7	12.6	10.3	10.2	12.9	10.2
OR	9.2	6.5	4.0	4.6	6.6	9.0
All sites	10.0	9.3	10.0	11.1	9.2	10.9
No. deaths/100,000 population						
All sites	2.2	1.9	2.3	2.2	2.2	2.6

*ABCs (Active Bacterial Core surveillance) areas: San Francisco, California (3 counties), Connecticut (entire state), Atlanta, Georgia, metropolitan area (20 counties), Baltimore, Maryland (6 counties), Minneapolis/St. Paul, Minnesota (7 counties), Rochester, New York (7 counties), and Portland, Oregon (3 counties).

### Demographic and Clinical Characteristics

In comparison to community-based case-patients, LTCF case-patients were older (median 83 years vs. 75 years for community case-patients, p<0.01) and more frequently female (Table 2). Underlying illness information was available for 1,538 (93%) case-patients. Congestive heart failure (CHF), diabetes mellitus, chronic obstructive pulmonary disease, and atherosclerotic cardiovascular disease were common in both groups. However, LTCF case-patients more frequently had CHF and a history of cerebrovascular accident but less commonly had diabetes mellitus or were current smokers than community-based case-patients. In addition, LTCF residents were less likely to have penetrating trauma preceding the infection (0.8% vs. 2.7%, p<0.05). Compared to community-based case-patients, LTCF case-patients more commonly had bacteremia without focus and pneumonia but less frequently had cutaneous or soft tissue infections as the possible source of the invasive GAS isolate identified (Table 3).

**Table 2:** Characteristics of persons age >65 y with invasive group A streptococcal infection by known residence, ABCs areas, 1998–2003*

Characteristic	No. LTCF case-patients (%), n = 383	No. community-based case-patients (%), n = 1,279	p value
Age, y			<0.01
65–74	72 (18.8)	584 (45.7)	
75–84	149 (38.9)	465 (36.3)	
>85	162 (42.3)	230 (18.0)	
Female sex	238 (62.1)	626 (48.9)	<0.01
Race†			0.16
White	282 (82.5)	914 (78.9)	
Black	50 (14.6)	182 (15.7)	
Other	10 (2.9)	63 (5.4)	
Case-fatality†	124 (32.6)	268 (21.1)	<0.01
Hospitalization†	346 (90.3)	1211 (94.8)	<0.01
Presence of underlying illnesses†			
Congestive heart failure	104 (29.3)	237 (20.5)	<0.01
Cerebrovascular accident	39 (16.8)	71 (9.4)	<0.01
Diabetes mellitus	86 (24.2)	346 (30.0)	<0.05
Current smoker	6 (2.1)	61 (6.5)	<0.01
Chronic obstructive pulmonary disease	62 (17.5)	172 (14.9)	0.24
Atherosclerotic cardiovascular disease	95 (26.7)	351 (30.4)	0.19
Renal failure/dialysis	30 (8.5)	103 (8.9)	0.78
Alcohol abuse	19 (5.4)	48 (4.2)	0.34
Immunosuppressive therapy‡	19 (5.4)	87 (7.5)	0.16

*ABCs, Active Bacterial Core surveillance;LTCF, long-term care facility. Case-patients with missing responses for residence type or individual characteristics were excluded from analysis. †Data were not available for all case-patients. Denominators by residence varied for the following: race (LTCF 342, community 1,159), outcome (LTCF 380, community 1,270), hospitalization (LTCF 383, community 1,278), underlying illnesses (LTCF 355, community 1,154) except for cerebrovascular accident (LTCF 232, community 758) and current smoker (LTCF 285, community 936). ‡Includes steroids, chemotherapy, and radiation therapy.

**Table 3:** Clinical syndromes among persons >65 y with invasive group A streptococcal infection, by residence and overall CFR, ABCs areas, 1998–2003*

Clinical syndrome	No. LTCF case-patients (%), N = 383	No. community-based case-patients (%), N = 1,279	p value	Overall CFR, %
Bacteremia without focus	145 (37.9)	406 (31.7)	<0.05	25.1
Pneumonia†	97 (25.3)	225 (17.6)	<0.01	34.0
Cellulitis†	121 (31.6)	498 (38.9)	<0.01	16.3
Septic arthritis†	20 (5.2)	90 (7.0)	0.21	11.8
Osteomyelitis†	7 (1.8)	26 (2.0)	0.80	6.1
STSS	15 (3.9)	82 (6.4)	0.07	55.7
Necrotizing fasciitis	15 (3.9)	80 (6.3)	0.08	36.6
Abscess†‡	8 (2.3)	47 (3.9)	0.15	14.5

*CFR, case-fatality ratio; ABCs, Active Bacterial Core surveillance; LTCF, long-term care facility; STSS, streptococcal toxic shock syndrome. Case-patients with missing responses for residence type, outcome, or clinical syndrome were excluded from analysis. Data for case**-**patients could be categorized under >1 syndrome except for case-patients identified as having bacteremia without a focus. †Occurring in conjunction with isolation of group A streptococcal infection from a sterile site (e.g., blood culture). ‡Data not available for all years. Denominators: LTCF 349; community 1,205.

### Isolate Characteristics

GAS was identified from blood cultures in 1,491 (90%) of the 1,662 elderly case-patients with known residence. Of the remaining 171 nonbacteremic patients, GAS was most commonly isolated from joint fluid (n = 57) and surgical specimens (n = 51). GAS was identified from multiple body sites in 125 (8%) case-patients.

GAS isolates were available in 1,414 (85%) of the 1,662 case-patients. From a total of 63 *emm* types identified, 5 (*emm1*, *emm3*, *emm12*, *emm28*, and *emm89*) accounted for most infections (57% among LTCF residents; 62% among community-based residents) (Table 4). Antimicrobial susceptibility testing was performed on 781 GAS isolates including 187 isolates from LTCF case-patients. Fourteen (7%) isolates from LTCF case-patients and 34 (6%) from community-based case-patients were not susceptible to erythromycin (p = 0.38). Three isolates from LTCF case-patients and 5 from community case-patients were not susceptible to levofloxacin; 2 from community case-patients were not susceptible to clindamycin. No isolates were resistant to penicillin, ampicillin, cefazolin, vancomycin, or cefotaxime.

**Table 4:** Most common *emm* types identified in persons >65 y with invasive group A streptococcal infection, by residence, ABCs areas, 1998–2003*

*emm* type	No. LTCF case-patients (%), N = 324	No. community-based case-patients (%), N = 1,090
1	55 (17.0)	233 (21.4)
3	44 (13.6)	141 (12.9)
28	39 (12.0)	122 (11.2)
12	21 (6.5)	116 (10.6)
89	27 (8.3)	61 (5.6)
77	9 (2.8)	39 (3.6)
6	12 (3.7)	22 (2.0)
18	6 (1.9)	28 (2.6)
11	10 (3.1)	23 (2.1)
4	11 (3.4)	21 (1.9)

*ABCs, Active Bacterial Core surveillance; LTCF, long-term care facility. Case-patients with missing responses for residence type and *emm* type were excluded from analysis. Table stratified by overall frequency.

### Predictors of Death

The CFR among case-patients >65 years of age was 24%. CFR increased with age among both LTCF- and community-based case-patients. However, when compared to the CFR for the 65- to 74-year-old group, the CFR among 75- to 84-year-old persons and those >85 years of age was significantly greater only among community-based case-patients (Figure). LTCF case-patients were 1.5 times as likely to die from the infection as community-based GAS case-patients (33% vs. 21%, p<0.01); however, this group was less often hospitalized (90% vs. 95%, p<0.01). CFRs among hospitalized and nonhospitalized case-patients were comparable in both LTCF (33% vs. 33%, p = 0.92) and community case-patients (21% vs. 25%, p = 0.44).

**Figure:**
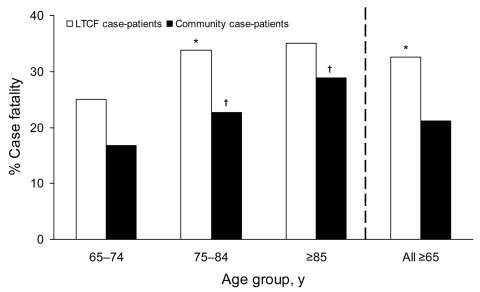
Comparison of case-fatality ratio from invasive group A streptococcal infections among persons by age group and residence, Active Bacterial Core surveillance areas, 1998–2003. Blank square, long-term care facility case-patient; black square, community-based case-patient. Case-patients with missing responses for residence type and outcomes were excluded from analysis. *p<0.05 for long-term care facility case-patients versus community-based case-patients. †p<0.05 indicates significance between the following groups: 75–84-year age group versus 65–74-year age group, or >85-year age group versus 65–74-year age group.

Univariate analysis of LTCF case-patients showed that those with CHF had significantly higher CFR (42% with CHF died vs. 27% without CHF, p<0.01) as did those with infections caused by *emm1* (51% vs. 28%, p<0.01) or *emm3* (45% vs. 30%, p<0.05) when compared to other *emm* types. We also observed higher CFR among LTCF case-patients with STSS (73% vs. 31%, p<0.01), NF (64% vs. 31%, p<0.05), or pneumonia (42% vs. 30%, p<0.05) than those with other syndromes. Sex, race, and hospitalization of LTCF case-patients were not significantly associated with death. These same variables were associated with significantly higher case-fatality rates among community-based case-patients.

In the final multivariate logistic regression model, independent predictors of death included LTCF residence; lack of hospitalization; infection due to *emm1*, *emm3*, or *emm12*; disease manifesting as STSS, NF, pneumonia, or bacteremia without focus; and interaction between female sex and presence of congestive heart failure (Table 5). Age was not a significant risk factor associated with death.

**Table 5:** Results of multivariate logistic regression analysis of factors associated with death from invasive group A streptococcal infection among case-patients >65 y of age, ABCs areas, 1998–2003*

Characteristic	Adjusted odds ratio (95% CI)
Age group, y	
>85	1.4 (0.9–2.1)
75–84	1.2 (0.8–1.8)
65–74	Reference
Race	
Black	0.8 (0.5–1.2)
Other than black	Reference
Residence	
**Long-term care facility**	**1.6 (1.1–2.2)**
Community	Reference
Hospitalized	
**Hospitalized**	**0.5 (0.3–0.9)**
Not hospitalized	Reference
Syndrome	
**Bacteremia without focus**	**2.6 (1.7–3.8)**
**Pneumonia**	**3.7 (2.4–5.8)**
**Necrotizing fasciitis**	**3.6 (1.7–7.4)**
**STSS**	**11.1 (6.4–19.3)**
Other syndrome	Reference
*emm* type	
***emm1***	**2.3 (1.4–3.6)**
***emm3***	**1.9 (1.1–3.1)**
*emm4*	1.7 (0.6–4.5)
*emm6*	0.6 (0.2–2.1)
*emm11*	0.4 (0.1–2.0)
***emm12***	**1.9 (1.1–3.4)**
*emm18*	1.3 (0.5–3.9)
*emm28*	0.9 (0.5–1.7)
*emm77*	1.3 (0.5–3.4)
*emm89*	1.5 (0.8–3.0)
Other *emm* types	Reference
Sex and history of CHF†	
**Females with CHF**	**2.4 (1.5–3.8)**
Females without CHF	0.9 (0.7–1.4)
Males with CHF	1.2 (0.7–2.0)
Males without CHF	Reference

*ABCs, Active Bacterial Core Surveillance; CI, confidence interval; STSS, streptococcal toxic shock syndrome; CHF, congestive heart failure. A total of 1,140 case-patients with complete data were included in the final model. Significant results are shown in **boldface**. †Interaction between sex and history of CHF.

### Clustering of Cases

We identified 18 GAS clusters comprising a total of 40 cases (10% of LTCF cases). Fourteen clusters consisted of only 2 cases; the other 4 clusters had 3 cases each. The median interval between the first and second cases was 2.5 months (range 0.2–9.2 months). The most common *emm* types identified were *emm28* and *emm89*, which caused 4 and 3 clusters, respectively. Case-patients in clusters were of similar age (median 85.5 years), sex (68% female), and race (75% white) to overall LTCF GAS case-patients >65 years of age. The most common syndromes of clustered patients were cellulitis (40%) and bacteremia without focus (38%). Fifteen case-patients died (CFR 38%).

## Discussion

Although the elderly have the highest rates of disease and death due to invasive GAS infection (*2*–*4*), we demonstrated that a subset of persons >65 years of age has an even greater risk. Invasive GAS infection was almost 6 times as likely to develop in elderly LTCF residents. Moreover, such case-patients were 1.5 times more likely to die from this infection than elderly persons living in the community. LTCF case-patients with invasive GAS infection were more likely to be older, female, have a history of CHF or CVA, and have pneumonia or bacteremia without focus compared to community-based case-patients. We found no significant differences in *emm* type distributions and antimicrobial resistance patterns among GAS isolates that caused infections in LTCF- or community-based case-patients.

The increased risk for death among elderly case-patients living in LTCFs compared to case-patients in the community remained significant on multivariate analysis and is likely attributable, in part, to the fact that LTCF residence is a proxy measure of individual frailty. While this surveillance system collects information such as age and underlying conditions, measurements of functional status such as the Karnofsky score or activities of daily living are not obtained. The common use of advanced directives among LTCF residents may also contribute to the higher CFR. Because some directives preclude aggressive clinical management, this may also explain the lower frequency of hospitalization among LTCF case-patients.

Other factors associated with higher CFR included specific *emm* types and several clinical syndromes. These findings are consistent with past studies in which disease due to *emm* types 1 and 3 as well as the clinical syndromes pneumonia or STSS were independent predictors of death among all age groups (*2*). Although advancing age has been found previously to contribute to overall case-fatality rates (*2*,*23*), our analysis showed advancing age (e.g., age 75–84 years or >85 years) was no longer significant once presence of CHF, residence type, and *emm* type were included in the statistical model.

The true extent of severe GAS infections in the LTCF population is likely greater than our study estimates. First, ABCs identifies only culture-confirmed invasive GAS infections, limiting recognition of GAS syndromes such as cellulitis, for which cultures are not commonly obtained. Furthermore, current guidelines developed through expert opinion do not recommend obtaining blood cultures in residents of LTCFs, largely because of the low yield of blood cultures in this setting (*24*). Consequently, many LTCF practitioners do not routinely obtain blood cultures in residents with fever; residents are either treated empirically or transferred to an acute-care facility (*25*,*26*). In our analysis of hospitalized LTCF case-patients, only 8% of positive GAS cultures were obtained before the day of hospitalization. Second, ABCs surveillance personnel have noted that residence-type is not always recorded in medical records, potentially leading to misclassification of LTCF residents as community residents. However, this misclassification would also underestimate the extent of severe GAS illness in the LTCF population.

We used available data to estimate the frequency of clusters of invasive GAS infection occurring in LTCFs. Although other studies suggest that many cases of invasive GAS may represent secondary transmission (*4*,*23*,*27*), we found that only 10% of cases among LTCF residents occurred within documented clusters. This finding likely represents underreporting for several reasons: use of empiric antimicrobial agents in LTCFs for mild and moderate infections; presence of disease manifestations for which cultures are not routinely obtained (e.g., cellulitis); and absence of GAS isolates (15%) for *emm* typing, a criterion we used to define a cluster.

Nonetheless, this study augments findings from other studies that note greater frequency of invasive bacterial infections among the elderly (*27*–*29*). Prior analyses of invasive group B streptococcal (GBS) and *S. pneumoniae* infections found that these infections were ≈4 times more common in LTCF residents than in community-dwelling elderly (*28*,*29*), likely due to the advanced age, multiple underlying conditions, and immobility in this population (*30*). Crowded living quarters may also play a role, as clusters of invasive GAS among healthy persons living in close proximity have been reported previously (*31*,*32*). Although less prevalent within nursing homes than illnesses such as urinary tract infection, invasive GAS, GBS, and pneumococcal diseases remain substantial causes for concern given the associated illness and higher deaths with these infections, the risk for outbreaks, and emerging antimicrobial resistance.

In addition to improved LTCF infection control practices, invasive GAS infections could be prevented with the use of an effective GAS vaccine. In the past, development of a GAS vaccine targeting the M protein, a major virulence determinant, has been halted over concerns of possible induction of antibodies that cross-react with brain, joint, and cardiac tissues (*33*,*34*). However, current vaccine candidates avoid the risks for cross-reactivity (*35*,*36*). Our analysis shows that 82% and 85% of strains causing invasive disease in both LTCF and community elderly, respectively, would be covered by the 26-valent M protein-based vaccine recently tested in phase II trials. If this vaccine also induces a protective response among older adults, it could substantially benefit LTCF residents.

In conclusion, our analysis noted that all older adults, but particularly those living in LTCFs, have significantly higher rates of disease and death from invasive GAS infection. This institutionalized population represents a unique opportunity for prevention through enhanced surveillance to improve case detection and secondary disease prevention, stringent infection control measures, and annual immunization against influenza, a disease for which GAS is a known secondary infection (*14*,*16*,*23*). Finally, vaccination of this population with an effective GAS vaccine may be highly beneficial.
